# Enhancing surgical instrument segmentation: integrating vision transformer insights with adapter

**DOI:** 10.1007/s11548-024-03140-z

**Published:** 2024-05-08

**Authors:** Meng Wei, Miaojing Shi, Tom Vercauteren

**Affiliations:** 1 School of Biomedical Engineering and Imaging Sciences, King’s College London, London, UK; 2 College of Electronic and Information Engineering, Tongji University, Shanghai, China

**Keywords:** Vision transformer, Adapter network, Surgical instrument segmentation

## Abstract

**Purpose:**

In surgical image segmentation, a major challenge is the extensive time and resources required to gather large-scale annotated datasets. Given the scarcity of annotated data in this field, our work aims to develop a model that achieves competitive performance with training on limited datasets, while also enhancing model robustness in various surgical scenarios.

**Methods:**

We propose a method that harnesses the strengths of pre-trained Vision Transformers (ViTs) and data efficiency of convolutional neural networks (CNNs). Specifically, we demonstrate how a CNN segmentation model can be used as a lightweight adapter for a frozen ViT feature encoder. Our novel feature adapter uses cross-attention modules that merge the multiscale features derived from the CNN encoder with feature embeddings from ViT, ensuring integration of the global insights from ViT along with local information from CNN.

**Results:**

Extensive experiments demonstrate our method outperforms current models in surgical instrument segmentation. Specifically, it achieves superior performance in binary segmentation on the Robust-MIS 2019 dataset, as well as in multiclass segmentation tasks on the EndoVis 2017 and EndoVis 2018 datasets. It also showcases remarkable robustness through cross-dataset validation across these 3 datasets, along with the CholecSeg8k and AutoLaparo datasets. Ablation studies based on the datasets prove the efficacy of our novel adapter module.

**Conclusion:**

In this study, we presented a novel approach integrating ViT and CNN. Our unique feature adapter successfully combines the global insights of ViT with the local, multi-scale spatial capabilities of CNN. This integration effectively overcomes data limitations in surgical instrument segmentation. The source code is available at: https://github.com/weimengmeng1999/AdapterSIS.git.

**Supplementary Information:**

The online version contains supplementary material available at 10.1007/s11548-024-03140-z.

## Introduction

Detecting and tracking surgical instruments in laparoscopic videos is crucial for autonomous surgery and enhanced clinical support [1]. The trend in the field is toward the utilization of deep learning methodologies [2, 3]. Current models heavily depend on fully supervised learning, requiring extensive annotated data. However, acquiring such data, especially in surgical tool segmentation, is expensive and time-intensive, resulting in the lack of large-scale annotated datasets, a significant hurdle for precise model development. Additionally, biases in training datasets arise from outdated datasets, geographical diversity, and unverified clinical relevance, affecting the robustness needed for applications like autonomous surgery.

In light of the rapid advancements in large-scale ViTs [4] and their excellent ability to learn from extensive data, pre-trained ViT models [4–6] offer promising potential for downstream tasks [7–9]. CNNs have revolutionized the medical image segmentation field. However, their localized convolution operations limit capturing global and long-range semantic interactions. Transformers provide global self-attention but might lack detailed localization abilities [10]. Merging CNN and ViT is a recent trend to leverage their strengths [10–13]. Yet, these methods, often starting from scratch, might not fully exploit pre-trained knowledge from large image datasets, a significant ViT strength. Moreover, while most of them focus on bridging the global and local information gaps between the two methods, they neglect the inherent advantages of each: CNNs exhibit better performance with limited datasets, whereas ViTs are superb with extensive data training.

Therefore, given the constraints imposed above, we harness the full potential of both ViT and CNN. We are particularly focused on capitalizing on the pre-trained general knowledge derived from ViT to enhance surgical image segmentation models, with an overarching goal of optimal both of the model performance and robustness within the complex and diverse domain of surgical images. Our main contributions are: (1) adapting a pre-trained and frozen ViT based on DINOV2 [6] to a CNN backbone segmentation model optimized for scenarios with limited annotated data; (2) introducing innovative adapter modules with cross-attention (CA) to integrate the global information from ViT and local features from CNN; (3) enhancing the generalizability of the segmentation model across multiple datasets.

## Related work

### Surgical instrument segmentation

The majority of surgical instrument segmentation works are CNN-based methods. For example, ISINet [3] proposes an instance-based surgical instrument segmentation CNN network that includes a temporal consistency module. OR-UNet [2] is introduced as an optimized 2D UNet [14] for instrument segmentation. There is a growing trend of exploring ViT-based methods. MATIS [15] is a fully transformer-based method that utilizes pixel-wise attention and masked attention modules. TraSeTR [16] introduces a track-to-segment transformer that leverages tracking cues to enhance surgical instrument segmentation.

### Pre-trained vision transformers

Driven by extensive pretraining on large datasets, ViT [4] and DINO [5] employs self-supervised learning for vision tasks. DINOV2 [6] improves DINO [5] by the training of large-scale ViT models with 1B parameters and distills it into smaller models. The pre-trained ViTs are successfully applied to the downstream tasks such as image classification [7, 8], object detection [6], semantic segmentation [6, 7], and video action classification [8]. Research on fine-tuning cross-attention modules with pre-trained embeddings [17] aligns with our method of harnessing pre-trained knowledge from large-scale ViT models. Yet, there is no existing work that adapts pre-trained ViT features by a CNN adapter, crucial due to limited data availability [4].

### Hybrid CNN and ViT models

ViTs and CNNs inherently complement each other. Numerous studies fuse two architectures to address their limitations. For instance, TransUNet [10] hybrids in which ViT processes CNN-derived patches for global context. TransFuse [11] parallels ViT and CNNs for efficient global and multi-level spatial feature fusion. There are also works that simulate the characteristics of CNN in their ViT models [8, 17] or directly adopt the cross-attention mechanism to augment the CNN structure [18], but none of the existing work integrates cross-attention into a CNN model to serve as a lightweight adapter for a pre-trained ViT model.

## Method

**Fig. 1:**
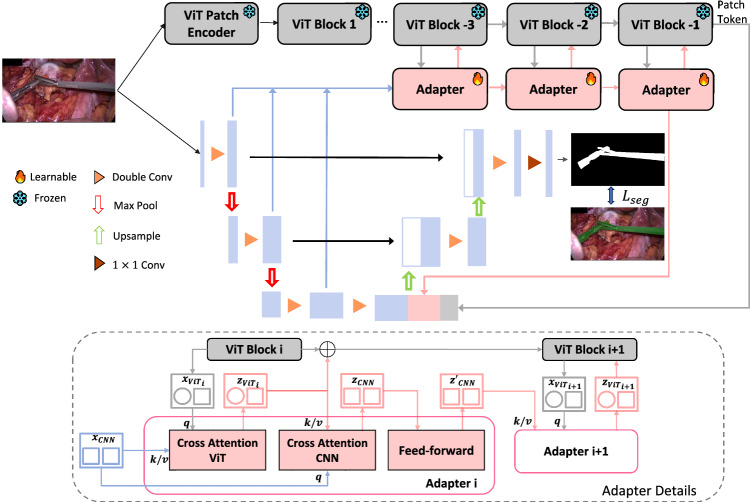
An overview of our method. Our model includes two main parts: the top consists of a frozen pre-trained ViT feature encoder; the middle introduces adapter modules that enable CA integration between multi-scale features from CNN and pre-trained ViT features; the bottom is backbone segmenter tailored for instrument segmentation; *q* is query and *k*/*v* is key/value

We present the three primary elements of our model with the detailed architecture illustrated in Fig. 1. The ViT feature encoder remains frozen, with only the adapter and the CNN backbone segmented undergoing training. The CNN decoder receives three distinct feature inputs: (1) patch tokens from the ViT branch, encapsulating local information; (2) output from the adapter, which combines local and global insights from both the ViT and CNN branches; (3) feature maps from the CNN encoder, preserving the spatial information of the original image.

### Vision transformer encoder

Our vision transformer encoder follows the established method inspired by ViT [4]. Given an input image, denoted as $I \in \mathbb {R}^{H \times W \times C}$, where *H* is the height and *W* is the width. The ViT encoder initially divides the image into patches, forming a sequence represented as $I = [I_1, \ldots , I_N] \in \mathbb {R}^{N \times P^2 \times C}$, where *P* corresponds to the patch size. The count of patches, *N*, is calculated as $N = \frac{\textrm{HW}}{{P^2}}$. Each of these individual patches is then converted into a 1D vector and linearly projected, resulting in a sequence of patch embeddings, denoted as $I_0 = [E_{I_1}, \ldots , E_{I_N}] \in \mathbb {R}^{N \times D}$, with the transformation matrix $E \in \mathbb {R}^{D \times (P^2C)}$. To account for positional information, the ViT encoder introduces learnable position embeddings to combine with the patch sequence. The transformer encoder then maps the input sequence of embedded patches with position encoding to the output $x_\textrm{ViT} = \left[ x_\textrm{ViT}^\textrm{patch} || x_\textrm{ViT}^\textrm{CLS}\right]$, a contextualized encoding sequence containing rich semantic information. To utilize pre-learned knowledge, we employed and froze the entire ViT model. However, we selectively integrated the ViT feature embeddings from the deeper layers into our backbone segmentation model using adapters. We opted not to utilize the shallower layers to optimize computational efficiency.

### Feature adapter

Building on the strengths of ViT and CNN highlighted in Sect. 1, our adapter integrates multi-scale features from the CNN backbone segmentation encoder with those from the pre-trained ViT feature encoder.

**Fig. 2:**
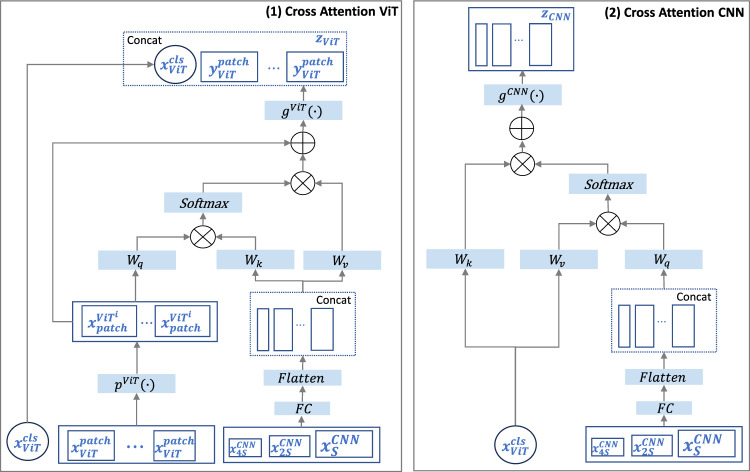
Cross-attention module for the ViT branch and backbone segmentation model: (1) Cross Attention ViT The feature embedding from CNN serves as a query to interact with the patch tokens from the ViT branch; (2) Cross Attention CNN The CLS token of the ViT serves as a query token to interact with the feature map from CNN through attention

*Cross attention for ViT* In our CA module for the ViT, we first utilize the patch token at the ViT branch, denoted as $x^\textrm{patch}_\textrm{ViT}$, which includes local information from the ViT pre-trained knowledge, as the query to exchange information among the multi-scale feature embeddings from the backbone segmentation encoder and then back project it to the ViT branch.

For visual clarity, Fig. 2 illustrates the CA module for ViT. Specifically, the multi-scale feature embeddings from the backbone segmenter encoder were initially aggregated. Several fully connected layers are applied at the end to project the feature maps to *D* dimensions, which equals the patch embedding size of the ViT branch. The multi-scale feature map from the backbone encoder, denoted as $x_{\textrm{HW}/S}$, $x_{\textrm{HW}/2S}$, and $x_{\textrm{HW}/4S}$, then comprises *D*-dimensional features at 1/*S*, 1/2*S*, and 1/4*S* resolutions of the original image, encompassing features with distinct receptive fields. Then we flatten and concatenate these feature maps, as illustrated in Eq.(1), serving as the key and value for the cross-attention, where || denotes the concatenation operation.

1 $$\begin{aligned} x_\textrm{CNN} = \text {Flatten}(\text {FC}(\left[ x_{\textrm{HW}/4S} || x_{\textrm{HW}/2S} || x_{\textrm{HW}/S}\right] )) \end{aligned}$$

Here, *S* represents the reduction scaling factor of the feature map size from the first layer of the backbone segmenter to the original input size. By taking $x_\textrm{ViT}^\textrm{patch}$, the module then performs CA between $x_\textrm{ViT}^\textrm{patch}$ and $x_\textrm{CNN}$. Mathematically, the CA can be expressed as:

$$\begin{aligned}{} & {} q = x_\textrm{ViT}^\textrm{patch}W_q, \quad k = x_\textrm{CNN}W_k, \quad v = x_\textrm{CNN}W_v,\\{} & {} A = \text {softmax}\left( \frac{qk^T}{\sqrt{D/h}}\right) , \quad CA(x_\textrm{CNN}) = Av \end{aligned}$$

where $W_q, W_k, W_v \in \mathbb {R}^{D \times (D/h)}$ are learnable parameters, *D* and *h* are the embedding dimension and number of heads. Specifically, the output of the CA for ViT module, denoted as $z_\textrm{ViT}$, is defined by the input from ViT and CNN branches with projection operations and residual shortcut as follows:

2 $$\begin{aligned}{} & {} y_\textrm{ViT}^\textrm{patch} = g^\textrm{ViT}(p^\textrm{ViT}(x_\textrm{ViT}^\textrm{patch}) + \text {CA}(x_\textrm{CNN})), \nonumber \\{} & {} z_\textrm{ViT}= x_\textrm{cls}^\textrm{ViT}||y_\textrm{ViT}^\textrm{patch} \end{aligned}$$

where $p^\textrm{ViT}(\cdot )$ and $g^\textrm{ViT}(\cdot )$ are projections to align dimensions.

*Cross attention for CNN* Our CA for CNN module is designed to facilitate information exchange between the global insights harnessed by the ViT branch and the localized details captured within the backbone segmentation encoder. The core mechanics of this process are akin to CA for ViT, albeit with a distinctive adjustment—here, the query and key/value roles are swapped.

More specifically, the multi-scale feature $x_\textrm{CNN}$ in Eq.(1) now takes on the role of the query. For the key and value, we exclusively utilize the CLS token of the ViT feature embedding. The CLS token has already assimilated abstract information across all patch tokens within the ViT branch, constituting a global representation. This CA procedure can be concisely expressed as follows:

$$\begin{aligned}{} & {} q = x_\textrm{CNN}W_q, \quad k = x_\textrm{ViT}^\textrm{CLS}W_k, \quad v = x_\textrm{ViT}^\textrm{CLS}W_v, \\{} & {} A = \text {softmax}\left( \frac{qk^T}{\sqrt{D/h}}\right) , \quad \textrm{CA}(x_\textrm{ViT}^\textrm{CLS}) = Av \end{aligned}$$

Note that the character definitions remain consistent with those in the CA for ViT module. Therefore, similar to the above, the output of the CA for CNN module with the residual shortcut can be defined as below:

3 $$\begin{aligned} z_\textrm{CNN} = g^\textrm{CNN}\left( x_\textrm{CNN} + \text {CA}\left( x_\textrm{ViT}^\textrm{CLS}\right) \right) \end{aligned}$$

where $g^\textrm{CNN}(\cdot )$ is the projection that aligns the dimension of the output feature map size to the input for the feed-forward network. This approach ensures that the size of the feature embeddings remains unchanged, while simultaneously amalgamating global insights from the ViT branch and local details from the CNN branch.

*Feed forward network* This module is a composite of key layers: convolution, activation, dropout for regularization, and a fully connected layer, working together to process and enhance the feature map to obtain $z'_\textrm{CNN}$. Their concerted efforts aim to extract vital features essential for the backbone segmentation model’s decoder.

*Data flow* The latter adapter takes the output from the previous adapter, $z'_\textrm{CNN}$, which has interacted with block *i* of the ViT branch, as its input for the subsequent CA for ViT module, engaging with the feature embedding $x_{\textrm{ViT}_{i+1}}$ from block $i+1$ of ViT. Note that the input of block $i+1$ is the sum of the output of CA for ViT in the previous adapter and the feature embedding $x_{\textrm{ViT}_i}$ of block *i*, denoted as $z_\textrm{ViT}$. The final output of the last adapter, interfaced with the final ViT block, serves as the input for the backbone segmentation decoder.

### Backbone segmentation model

For our backbone segmentation model, we use the UNet-like [14] structure. The encoder is constructed as a series stride-2 3$\times$3 convolutions and MaxPooling layers. The feature maps from each layer of the encoder are contacted to create multi-scale feature maps, subsequently fed into the adapter as shown in Sect. 3.2.

For the input to the backbone decoder, the ultimate feature map from the CNN encoder is combined with the output of the adapter which encompasses global insights from the ViT branch and local information from the CNN branch. Additionally, the patch tokens of the final feature embedding from the ViT branch were also contacted to preserve the contextual information of ViT.

Our backbone decoder is designed with a sequence of upsampling and convolutional layers. Significantly, we implement skip connections, a key feature that links feature maps at corresponding scales from the encoder to the decoder.

### Implementation details

*Loss function* In surgical image datasets, a substantial number of images predominantly comprise a background with no visible tools. Even in cases where tools are present, they often occupy a relatively small portion of the overall image. To address the class imbalance, we combine the Dice Loss with the Focal Tversky Loss for the assessment of our predictions against the ground truth segmentation map.

*Model configuration* We construct our ViT feature encoder in three distinct sizes, denoted as ViT-T, ViT-S, ViT-B, and ViT-g, all pre-trained using the DINOV2 framework [6]. These models exhibit varying parameter counts for our adapters: 21 M, 86 M, 14.0M, and 300 M, respectively. The number of attention heads is configured as 6, 6, and 12. In our setup, we chose a patch size of 14, resulting in a feature map scale of 1/14 for the ViT models. Additionally, for the CNN branch, the scaling factor *S* is set to 2, effectively leading to multi-scale feature maps with scales of 1/4, 1/8, and 1/16.

*Hyper parameters* The input image is 588$\times$588, considering the ViT branch’s input requirement, and augmented with the image augmentation techniques presented in [4]. We adopt the SGD optimizer with a learning rate of 0.01 and momentum of 0.9. We applied the linear scaling rule to reduce the learning rate. The model is trained on 2 V100 GPUs, and the batch size is set to 16.

## Experiments

### Datasets and evaluation metrics

*Datasets* Our binary segmentation experiments on the Robust-MIS 2019 [1] dataset utilized 5,983 annotated images for training, with three-stage testing, where stage 3 is from a procedure unseen during training. Multi-class segmentation was performed on EndoVis 2017 [19] and EndoVis 2018 [20]. Cross-dataset validation was conducted across the aforementioned datasets, along with CholecSeg8k [21] and AutoLaparo [22]. Each dataset was split into training and validation subsets at an 8:2 ratio with no patient overlap across folds.

*Evaluation metrics* For the state-of-the-art comparison experiments on binary segmentation, we assessed our model using the metrics outlined in the Robust-MIS 2019 challenge [1], which includes Dice Similarity Coefficient and Normalized Surface Dice (NSD) [1]. Following the challenge’s specifications [1], we adopted a 13-pixel tolerance for NSD. For the cross-dataset validation and ablation study, we also use the mean Intersection over Union (mIoU). For multi-class segmentation, we applied Ch_IoU, ISI_IoU, and mc_IoU following the evaluation metrics provided in [3, 23].

### Results

*Comparison to state-of-the-art* In Table 1, we compare our model with several state-of-the-art models on Robust-MIS 2019 dataset for binary segmentation. Our model outperformed the CNN models designed for this task and the pre-trained ViT models for natural semantic segmentation downstream, indicating the success of merging the pre-trained knowledge with the CNN models. The existing hybrid approaches were trained for a shorter duration (smaller epochs) which signifies a potential for improvement. An essential takeaway here is that our proposed model exhibits superior efficiency: it requires minimal training to yield outstanding outcomes.

**Table 1:** Comparison on the Robust-MIS 2019 dataset between state-of-the-art models: above are the fully supervised CNN and ViT models for surgical segmentation task; the middle is the existing hybrid CNN-ViT models (all trained for 400 epochs); the bottom is the pre-trained ViT model for semantic segmentation downstream

Method	Whole testing		Stage 1		Stage 2		Stage 3	
	Mean dice	NSD	Mean dice	NSD	Mean dice	NSD	Mean dice	NSD
OR-Unet [2]	88.0	86.2	90.2	88.5	87.9	85.6	85.9	84.5
Robust-MIS 2019 winner [1]	90.1	88.9	92.0	92.7	90.2	88.6	89.0	86.4
ISINet [3]	88.9	86.3	90.9	87.6	89.6	86.5	86.2	84.7
TransUNet[10]	79.6	76.5	82.2	77.9	80.4	76.2	75.2	75.4
TransFuse [11]	80.1	78.6	82.2	79.1	81.3	79.0	76.8	77.7
Swin TransV2 [8]	82.9	78.6	84.6	80.2	84.0	79.9	80.1	75.7
MaskFormer [7]	84.1	80.5	87.2	84.3	85.9	80.2	79.2	77
Ours	92.9	91.5	94.2	92.4	92.6	91.4	91.9	90.7

For the multi-class segmentation task, we also compare our model with existing models including S3Net[23], TraSeTR[16], and MSLRGR [24]. Table 2 shows our model outperforms the state-of-the-art on the EndoVis 2018 dataset with +15.78 percentage point (pp) gain in mc_IoU. The improvements across both datasets demonstrate the multi-class segmentation capability of our model. Moreover, our model outperforms MSLRGR [24], which directly introduces global context into CNN, suggesting our approach of integrating the global information from pre-trained ViT is more effective than the state-of-the-art models.

**Table 2:** Table 2 Comparison of our method with state-of-the-art methods on the EndoVis 2017 and EndoVis 2018 datasets for multi-class segmentation

Method	Ch_IoU	ISI_IoU	Bipolar	Prograsp	Large	Vessel	Grasping	Monopolar	Ultrasound	mc_IoU
			Forceps	Forceps	Needle driver	Instrument	Applier	Curved scissors	Probe	
EndoVis 2017										
TraSeTR [16]	60.40	65.20	45.20	56.70	55.80	38.90	11.40	31.3	18.20	36.79
S3Net [23]	72.54	**71**.**99**	**75**.**08**	54.32	61.84	35.5	**27**.**47**	**43**.**23**	28.38	46.55
Ours	**73**.**96**	69.15	66.45	**67**.**56**	**70**.**52**	**42**.**68**	12.9	40.15	**29**.**12**	**47**.**06**
EndoVis 2018										
TraSeTR [16]	76.20	–	76.30	53.30	46.50	40.60	13.90	86.30	17.50	47.77
S3Net [23]	75.81	74.02	77.22	50.87	19.83	50.59	0.00	**92**.**12**	7.44	42.58
MSLRGR [24]	–	–	69.66	43.56	0.15	34.71	3.87	87.16	12.03	35.88
Ours	**85**.**25**	**82**.**99**	**85**.**72**	**67**.**86**	**72**.**56**	**89**.**16**	**6**.**39**	91.07	**22**.**12**	**63**.**55**

The values in bold signifies the best performance in the specific metric represented by that column

*Cross dataset validation* We conducted experiments using a cross-dataset validation approach, where we trained the model on one dataset and validated it on another, shown in Table 3. We present comparative experiments between our model, the top-performing CNN model OR-Unet [2], and ViT-based model MaskFormer [7].

**Table 3:** Cross dataset validation on EndoVis 2017, EndoVis 2018, CholecSeg8k, Robust-MIS 2019, and AutoLaparo datasets for OR-Unet [2], MaskFormer [7], and our method

Train dataset	Model	Test Dataset									
		EndoVis 2017		EndoVis 2018		CholecSeg8k		Robust-MIS 2019		AutoLaparo	
		Mean dice	mIoU	Mean dice	mIoU	Mean dice	mIoU	Mean dice	mIoU	Mean dice	mIoU
EndoVis 2017	OR-UNet [2]	92.4	81.3	73.0	62.4	74.3	65.7	59.2	10.3	74.5	56.7
	MaskFormer [7]	93.2	84.2	79.8	70.2	73.8	65.2	54.2	19.7	83.2	52.8
	Ours	98.9	96.2	94.2	85.8	85.9	80.7	88.4	80.6	89.9	69.7
EndoVis 2018	OR-UNet [2]	85.1	64.2	89.5	77.9	68.2	64.3	57.4	12.9	76.9	52.8
	MaskFormer [7]	84.3	72.2	88.2	81.8	74.8	61.9	56.7	31.9	77.9	65.9
	Ours	98.1	89.5	94.9	86.2	86.2	81.5	84.5	63.2	90.4	83.9
CholecSeg8k	OR-UNet [2]	82.3	71.4	69.9	53.2	82.7	75.4	51.5	8.2	69.7	61.4
	MaskFormer [7]	80.1	70.2	78.7	69.9	86.9	80.7	52.9	20.3	72.9	62.2
	Ours	95.9	88.6	92.1	82.8	91.9	86.6	90.1	83.5	90.2	82.4
Robust-MIS 2019	OR-UNet [2]	73.6	45.5	70.8	59.2	67.6	55.2	88.0	86.2	65.1	62.5
	MaskFormer [7]	86.4	79.0	81.8	70.1	77.2	62.7	84.1	80.5	71.8	65.2
	Ours	97.9	91.4	93.2	84.5	86.5	70.2	92.9	86.6	95.1	89.5
AutoLaparo	OR-UNet [2]	71.9	65.2	69.1	52.7	62.7	43.1	62.1	31.4	82.1	75.3
	MaskFormer [7]	85.1	73.6	79.0	60.8	76.4	63.2	60.5	37.4	92.7	84.9
	Ours	97.2	89.9	91.8	81.2	89.2	84.6	91.6	83.7	96.9	92.3

OR-UNet [2] and MaskFormer [7] experience significant performance drops when the training and testing datasets are different, while these variations are substantially reduced when they are trained and tested on the same dataset, yet the performance variability underscores their limited generalizability. Conversely, our model maintains consistent scores across different datasets, indicating its excellent robustness and accuracy. Some combinations, like training on EndoVis 2017 and testing on Robust-MIS 2019, show a more significant drop in performance than others, which hints at challenges the model faces when trained on a comparatively simpler dataset and tested on more complex, real-world data (Table 3).

### Ablation study

*Transformer feature encoder* In Table 4, we conducted an ablation on the transformer feature encoder, and observed utilizing only the last layer resulted in a notable drop in both Dice scores and mIoU across datasets. However, by incorporating the last 3 layers, we observed performance metrics are close to that using all layers. Importantly, this configuration with the last 3 layers strikes a balance, offering near-optimal performance while being significantly more computationally efficient.

**Table 4:** Ablation studies on the transformer encoder when trained on Robust-MIS 2019 and tested on Robust-MIS 2019 and cross-dataset validated on CholecSeg8k dataset

Transformer encoder	Robust-MIS 2019		CholecSeg8k	
	Dice	mIoU	Dice	mIoU
All blocks	93.2	87.1	87.4	71.5
Last block	88.9	83.2	83.7	67.5
Last 3 blocks (ours)	92.9	86.6	86.5	70.2

**Table 5:** Ablation studies for adapter when trained on Robust-MIS 2019 and tested on Robust-MIS 2019 and cross-dataset validated on CholecSeg8k dataset

Adapter		Robust-MIS 2019		CholecSeg8k	
		Dice	mIoU	Dice	mIoU
$\times$ CA ViT &CNN		85.3	76.9	81.2	66.5
$\times$ CA ViT		88.9	80.1	82.6	65.7
$\times$ CA CNN		89.8	83.4	83.9	68.2
CA ViT	Single scale	89.5	81.9	83.2	68.2
	Patch $\rightarrow$ CLS	89.9	82.2	83.9	69.3
	$\times$ Residual	90.6	85.4	84.1	70.5
CA CNN	Single scale	91.3	87.4	82.7	68.6
	CLS $\rightarrow$ Patch	89.2	81.5	82.6	70.1
	$\times$ Residual	91.6	86.9	85.5	69.3
	Ours	92.9	86.6	86.5	70.2

*Adapter* We conduct the ablation study with or without CA for ViT and CA for CNN as shown in Table 5. When CA modules are removed entirely, there’s a substantial decrease in Dice and mIoU scores, highlighting their importance to the model’s performance and robustness. The drop is less severe when CA is removed only for CNN, suggesting the importance of integrating patch tokens from the pre-trained ViT embeddings.

*Cross attention for ViT* We offer the ablation study for the adapter module in Table 5. For the CA for ViT module, we observe that (1) when solely relying on the single scale, there was a decrease of 3.4 pp in Dice scores on Robust-MIS 2019, indicating the significance of multi-scale features in capturing diverse spatial information; (2) adopting the strategy of replacing the patch tokens with CLS token has led to some performance decreases, suggesting incorporating the global information from the CLS token, loses the local details that patch tokens offer; (3) excluding the shortcut residuals leads to a drop in the Dice score by 2.3 pp for Robust-MIS 2019 gave the importance of residual shortcut to maintain information flow; (4) even with variations in the ablation studies causing some drops in performance, the model’s consistent decent scores on CholecSeg8k, underscores its superb generalization capability across datasets.

*Cross attention for CNN* In the context of the CA for CNN module shown in Table 5, using only the single scale results in a lesser decline in Dice score compared to that in CA for ViT, which suggests the output of CA for ViT already embodies multi-scale information, reducing its impact for the latter CA for CNN module. Opting to substitute the CLS token with patch tokens, despite being computationally costly, has observed a decrease in performance. This highlights the significance of integrating global information within the CNN branch.

## Conclusion

In conclusion, our research presents an innovative approach to surgical image segmentation by combining ViT with a CNN used as a lightweight adapter module. Our work tackles the challenge of gathering large-scale annotated data and enhances the generalizability of different surgical scenarios. Our unique feature adapter, integrating cross-attention modules, facilitates the fusion of global and local, multi-scale spatial information from ViT and CNN, respectively. Our model achieves excellent accuracy and robustness across diverse surgical scenarios, as evidenced by our model’s superior performance on the Robust-MIS 2019 dataset and across five other datasets. Our model has potential for applications in autonomous surgery, offering a solution that is both robust and adaptable to varying surgical environments.

### Supplementary Information

Below is the link to the electronic supplementary material.

Supplementary file 1 (pdf 516 KB)
