# Supplementary material for: Enhancing surgical instrument segmentation: integrating vision transformer insights with adapter
Source: Int J Comput Assist Radiol Surg. 2024 May 8;19(7):1313–20. doi: 10.1007/s11548-024-03140-z (PMC11230947; doi:10.1007/s11548-024-03140-z)
Supplement: Supplementary file 1 — (pdf 516 KB) [file 11548_2024_3140_MOESM1_ESM.pdf]

Supplementary of Enhancing Surgical Instrument Segmentation: Integrating Vision Transformer Insights with Adapter

Meng Wei[1*], Miaojing Shi[2] and Tom Vercauteren[1]

[1*]School of Biomedical Engineering & Imaging Sciences, King's College London, London, United Kingdom.
[2]College of Electronic and Information Engineering, Tongji University , Shanghai, China.

*Corresponding author(s). E-mail(s): meng.wei@kcl.ac.uk;
Contributing authors: mshi@tongji.edu.cn; tom.vercauteren@kcl.ac.uk;

1 Qualitative Results

Figure 1 displays representative samples for the binary segmentation on Robust-MIS 2019 dataset across various performance scenarios, which shows our model demonstrates well-delineated edges, manages overlapping instruments, and ensures the detection of details. In the cases that have the lowest Mean Dice scores, there is boundary ambiguity in high-reflection scenarios.

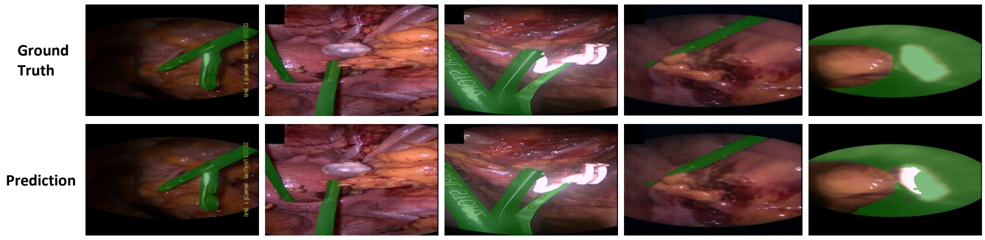

Fig. 1 Visualization of our model's binary segmentation predictions alongside ground truth: the first two columns on the left represent cases with the highest Mean Dice scores; the central columns highlight specific examples of surgical tools' intersection and discrete scenarios; the rightmost column depicts cases with the lowest Mean Dice score

Figure 2 presents a range of performance scenarios for multi-class segmentation on the EndoVis 2017 dataset. Our model demonstrates strong classification and segmentation capabilities, precisely recognizing classes with well-defined boundaries and accurate shapes. However, some challenges persist, particularly when certain instrument classes are near boundaries or partially obscured, leading to incomplete recognition of their shapes.

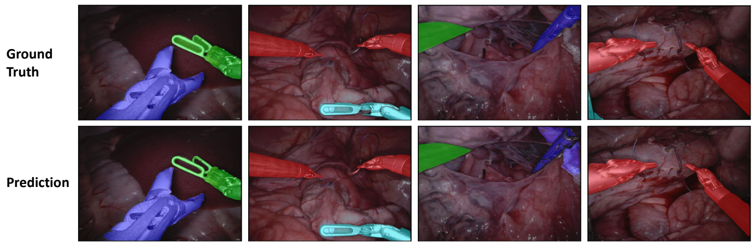

Fig. 2 Visualization of our multi-class segmentation prediction alongside the ground truth: The left-most two cases exhibit the highest Ch_IoU scores, while the second case from the right demonstrates the lowest Ch_IoU. The most leftward case indicates complete failure in recognizing one class.

2 Training Memory

We report the training memory for all blocks and the number of parameters of our model when employing ViT-S and ViT-B as the feature encoder for the ViT branch, as detailed below.

Table 1 Training Memory and Parameter Count for Our Model

Model	Training Memory (GB)	Parameters (Millions)
Ours with ViT-S	16.5 GB	69 M
Ours with ViT-B	25 GB	136 M

3 Statistical Significance

We performed a t-test using the data presented in Table 1 (Comparison on the Robust-MIS 2019 dataset between state-of-the-art models) in our manuscript to statistically validate the significance of our model's improvements. Sample groups were chosen from the Robust-MIS 2019 dataset, and we calculated the p-values to compare our model's performance against the current state-of-the-art methods. The p-value of our model against the next best-performing model indicates statistical significance at a standard 0.05 level.

4 Comprehensive Related Work

4.1 Surgical Instrument Segmentation

The majority of surgical instrument segmentation works are CNN-based methods. For example, ISINet [1] proposes an instance-based surgical instrument segmentation CNN network that includes a temporal consistency module to leverage temporal information. OR-UNet [2] is introduced as an optimized and robust 2D UNet [3] with residual blocks and multi-scale segmentation maps for instrument segmentation in endoscopic images. Although the majority of approaches rely on CNNs, there is a growing trend of exploring vision transformer-based methods in recent works. For instance, MATIS [4] is a fully transformer-based method that utilizes pixel-wise attention mechanisms and a masked attention module for surgical instrument segmentation while enhancing temporal consistency with video transformers. TraSeTR [5] introduces a Track-to-Segment transformer that leverages tracking cues, prior temporal knowledge, and contrastive query learning to enhance surgical instrument segmentation.

4.2 Pre-trained Vision Transformers

Driven by extensive pretraining on large datasets, ViT [6] employs masked patch prediction for self-supervised vision tasks. He *et al.* [7] presents masked autoencoders (MAE) for efficient self-supervised vision learning, using an asymmetric encoder-decoder and heavy input masking. Similarly, BEiT [8] advances the notion of predicting discrete tokens within vision models. Furthermore, DINO [9] investigates whether self-supervised learning imparts unique advantages to ViT architectures, particularly in terms of enhancing semantic segmentation capabilities. DINOV2 [10] continues to advance and enhance the training of large-scale ViT models with 1B parameters and distils it into a series of smaller models, surpassing the best available all-purpose features. The pre-trained vision transformers are successfully applied to the downstream tasks such as image classification [11, 12], object detection [10], semantic segmentation [10, 11], and video action classification [12]. Research on fine-tuning cross-attention modules with pre-trained embeddings, such as in [13], aligns with our method of harnessing pre-trained knowledge from large-scale ViT models. Yet, there is no existing work that adapts pre-trained ViT features by a CNN adapter, crucial due to limited data availability [6].

4.3 Hybrid CNN and ViT Models

ViTs and CNNs inherently complement each other. The Swin transformer network [12] adapts the concept of an expanding receptive field from CNNs for use in ViT There are also numerous studies that advocate for the fusion of these two architectures to address the limitations of each model. For instance, TransUNet [14] hybrids in which ViT processes CNN-derived patches for global context and the decoder combines these with high-resolution CNN maps for diverse medical applications. By employing a parallel architecture that combines ViT and CNNs, TransFuse [15] efficiently captures global dependencies and low-level spatial details, featuring the novel BiFusion module for effective multi-level feature fusion. Recently, CTCNet [16] was designed for medical

image segmentation, which combines Swin Transformers [17] and Residual CNNs using a cross-domain Fusion Block. There are also works that simulate the characteristics of CNN in their ViT models [13] or directly adopt the cross-attention mechanism to augment the CNN structure [18], but none of the existing work integrates cross-attention into a CNN model to serve as a lightweight adapter for a pre-trained ViT model.

5 Detailed Datasets Introduction

We conducted our main evaluating experiments on Robust-MIS 2019 dataset, comprising 10,040 annotated endoscopic images from 30 surgeries [19]. The training data involved 5,983 video clips from the proctocolectomy procedure and rectal resection procedure, with each clip's final frame annotated. Testing was structured in three stages: Stage 1 and 2 included 1,177 images, and Stage 3 introduced 2,880 images from the sigmoid resection, a procedure not present in the training set.

We also performed cross-dataset validation within Robust-MIS 2019 dataset and other 4 surgical image datasets, including 1) EndoVis 2017 [20] which features robotic instrument images from robotic-assisted surgeries [16]. The training set utilized 225 frames from 8 sequences, annotated for tool details. Testing involved the last 75 frames from these 8 sequences plus 2 full-length sequences (300 frames each) distinct from training; 2) EndoVis 2018 [21] that includes 15 video sequences, divided into 11 for training and 4 for testing, encompassing 7 specified instrument types; 3) CholecSeg8k [22] consists of 80 cholecystectomy surgery videos and each video provides 80 annotated frames summing up to 8,080 frames across 101 directories. 4) AutoLaparo [23] is derived from full-length hysterectomy videos, resulting in a segmentation sub-dataset of 1,800 frames. Each dataset was split into training and validation subsets at an 8:2 ratio with no patient overlap across folds.

References

[1] González, C., Bravo-Sánchez, L., Arbelaez, P.: Isinet: an instance-based approach for surgical instrument segmentation. In: MICCAI, pp. 595–605 (2020). Springer

[2] Isensee, F., Maier-Hein, K.: Or-unet: an optimized robust residual u-net for instrument segmentation in endoscopic images. ArXiv **abs/2004.12668** (2020)

[3] Ronneberger, O., Fischer, P., Brox, T.: U-net: Convolutional networks for biomedical image segmentation. In: MICCAI, pp. 234–241 (2015). Springer

[4] Ayobi, N., Pérez-Rondón, A., Rodríguez, S., Arbeláez, P.: Matis: Masked-attention transformers for surgical instrument segmentation. ISBI, 1–5 (2023)

[5] Zhao, Z., Jin, Y., Heng, P.-A.: Trasetr: track-to-segment transformer with contrastive query for instance-level instrument segmentation in robotic surgery. In: ICRA, pp. 11186–11193 (2022). IEEE

[6] Dosovitskiy, A., Beyer, L., Kolesnikov, A., Weissenborn, D., Zhai, X., Unterthiner, T., Dehghani, M., Minderer, M., Heigold, G., Gelly, S.: An image is worth 16x16 words: Transformers for image recognition at scale. ICLR (2021)

[7] He, K., Chen, X., Xie, S., Li, Y., Dollár, P., Girshick, R.: Masked autoencoders are scalable vision learners. In: Proceedings of the IEEE/CVF Conference on Computer Vision and Pattern Recognition, pp. 16000–16009 (2022)

[8] Bao, H., Dong, L., Piao, S., Wei, F.: BEiT: BERT Pre-Training of Image Transformers (2022)

[9] Caron, M., Touvron, H., Misra, I., Jégou, H., Mairal, J., Bojanowski, P., Joulin, A.: Emerging properties in self-supervised vision transformers. In: ICCV, pp. 9650–9660 (2021)

[10] Oquab, M., Darcet, T., Moutakanni, T., Vo, H., Szafraniec, M., Khalidov, V., Fernandez, P., Haziza, D., Massa, F., El-Nouby, A.: Dinov2: Learning robust visual features without supervision. arXiv preprint arXiv:2304.07193 (2023)

[11] Cheng, B., Schwing, A., Kirillov, A.: Per-pixel classification is not all you need for semantic segmentation. NeurIPS **34**, 17864–17875 (2021)

[12] Liu, Z., Hu, H., Lin, Y., Yao, Z., Xie, Z., Wei, Y., Ning, J., Cao, Y., Zhang, Z., Dong, L., Wei, F., Guo, B.: Swin transformer v2: Scaling up capacity and resolution. In: CVPR, pp. 12009–12019 (2022)

[13] Gheini, M., Ren, X., May, J.: On the strengths of cross-attention in pretrained transformers for machine translation. ArXiv **abs/2104.08771** (2021)

[14] Chen, J., Lu, Y., Yu, Q., Luo, X., Adeli, E., Wang, Y., Lu, L., Yuille, A.L., Zhou, Y.: Transunet: Transformers make strong encoders for medical image segmentation. arXiv preprint arXiv:2102.04306 (2021)

[15] Zhang, Y., Liu, H., Hu, Q.: Transfuse: Fusing transformers and cnns for medical image segmentation. In: MICCAI, pp. 14–24 (2021). Springer

[16] Yuan, F., Zhang, Z., Fang, Z.: An effective cnn and transformer complementary network for medical image segmentation. Pattern Recognition **136**, 109228 (2023)

[17] Cao, H., Wang, Y., Chen, J., Jiang, D., Zhang, X., Tian, Q., Wang, M.: Swin-unet: Unet-like pure transformer for medical image segmentation. In: European Conference on Computer Vision, pp. 205–218 (2022). Springer

[18] Liu, M., Yin, H.: Cross attention network for semantic segmentation. In: ICIP, pp. 2434–2438 (2019). IEEE

[19] Ross, T., Reinke, A., Full, P.M., Wagner, M., Kenngott, H., Apitz, M., Hempe, H., Filimon, D.M., Scholz, P., Tran, T.N.: Robust medical instrument segmentation

challenge 2019. arXiv preprint arXiv:2003.10299 (2020)

[20] Allan, M., Shvets, A., Kurmann, T., Zhang, Z., Duggal, R., Su, Y.-H., Rieke, N., Laina, I., Kalavakonda, N., Bodenstedt, S.: 2017 robotic instrument segmentation challenge. arXiv preprint arXiv:1902.06426 (2019)

[21] Allan, M., Kondo, S., Bodenstedt, S., Leger, S., Kadkhodamohammadi, R., Luengo, I., Fuentes, F., Flouty, E., Mohammed, A., Pedersen, M.: 2018 robotic scene segmentation challenge. arXiv preprint arXiv:2001.11190 (2020)

[22] Hong, W.-Y., Kao, C.-L., Kuo, Y.-H., Wang, J.-R., Chang, W.-L., Shih, C.-S.: Cholecseg8k: a semantic segmentation dataset for laparoscopic cholecystectomy based on cholec80. arXiv preprint arXiv:2012.12453 (2020)

[23] Wang, Z., Lu, B., Long, Y., Zhong, F., Cheung, T.-H., Dou, Q., Liu, Y.: Autolaparo: A new dataset of integrated multi-tasks for image-guided surgical automation in laparoscopic hysterectomy. In: MICCAI, pp. 486–496 (2022). Springer
